# Sepsis Disrupts Mitochondrial Function and Diaphragm Morphology

**DOI:** 10.3389/fphys.2021.704044

**Published:** 2021-09-07

**Authors:** Thamires Siqueira Oliveira, Anderson Teixeira Santos, Cherley Borba Vieira Andrade, Johnatas Dutra Silva, Natália Blanco, Nazareth de Novaes Rocha, Juliana Woyames, Pedro Leme Silva, Patricia Rieken Macedo Rocco, Wagner Seixas da-Silva, Tânia Maria Ortiga-Carvalho, Flavia Fonseca Bloise

**Affiliations:** ^1^Laboratory of Translational Endocrinology, Carlos Chagas Filho Institute of Biophysics, Federal University of Rio de Janeiro, Rio de Janeiro, Brazil; ^2^Laboratory of Metabolic Adaptations, Institute of Medical Biochemistry Leopoldo de Meis, Federal University of Rio de Janeiro, Rio de Janeiro, Brazil; ^3^Laboratory of Pulmonary Investigation, Carlos Chagas Filho Institute of Biophysics, Federal University of Rio de Janeiro, Rio de Janeiro, Brazil; ^4^Physiology and Pharmacology Department, Biomedical Institute, Fluminense Federal University, Niteroi, Brazil; ^5^Laboratory of Molecular Endocrinology, Carlos Chagas Filho Institute of Biophysics, Federal University of Rio de Janeiro, Rio de Janeiro, Brazil

**Keywords:** diaphragm, sepsis, mitochondria, CLP, oxidative phosphorylation, muscle

## Abstract

**Background:**

The diaphragm is the primary muscle of inspiration, and its dysfunction is frequent during sepsis. However, the mechanisms associated with sepsis and diaphragm dysfunction are not well understood. In this study, we evaluated the morphophysiological changes of the mitochondrial diaphragm 5 days after sepsis induction.

**Methods:**

Male C57Bl/6 mice were divided into two groups, namely, cecal ligation and puncture (CLP, *n* = 26) and sham-operated (*n* = 19). Mice received antibiotic treatment 8 h after surgery and then every 24 h until 5 days after surgery when mice were euthanized and the diaphragms were collected. Also, diaphragm function was evaluated *in vivo* by ultrasound 120 h after CLP. The tissue fiber profile was evaluated by the expression of myosin heavy chain and SERCA gene by qPCR and myosin protein by using Western blot. The *Myod1* and *Myog* expressions were evaluated by using qPCR. Diaphragm ultrastructure was assessed by electron microscopy, and mitochondrial physiology was investigated by high-resolution respirometry, Western blot, and qPCR.

**Results:**

Cecal ligation and puncture mice developed moderated sepsis, with a 74% survivor rate at 120 h. The diaphragm mass did not change in CLP mice compared with control, but we observed sarcomeric disorganization and increased muscle thickness (38%) during inspiration and expiration (21%). Septic diaphragm showed a reduction in fiber myosin type I and IIb mRNA expression by 50% but an increase in MyHC I and IIb protein levels compared with the sham mice. Total and healthy mitochondria were reduced by 30% in septic mice, which may be associated with a 50% decrease in *Ppargc1a* (encoding PGC1a) and *Opa1* (mitochondria fusion marker) expressions in the septic diaphragm. The small and non-functional OPA1 isoform also increased 70% in the septic diaphragm. These data suggest an imbalance in mitochondrial function. In fact, we observed downregulation of all respiratory chain complexes mRNA expression, decreased complex III and IV protein levels, and reduced oxygen consumption associated with ADP phosphorylation (36%) in CLP mice. Additionally, the septic diaphragm increased proton leak and downregulated *Sod2* by 70%.

**Conclusion:**

The current model of sepsis induced diaphragm morphological changes, increased mitochondrial damage, and induced functional impairment. Thus, diaphragm damage during sepsis seems to be associated with mitochondrial dysfunction.

## Graphical Abstract

Septic diaphragm has impaired morphology and increased thickness that seems to be associated, at least in part, with decreased mitochondrial function related to reducing in *Pgc1*α expression, ATP production, mitochondrial number, and quality in the CLP mice compared with the control group.

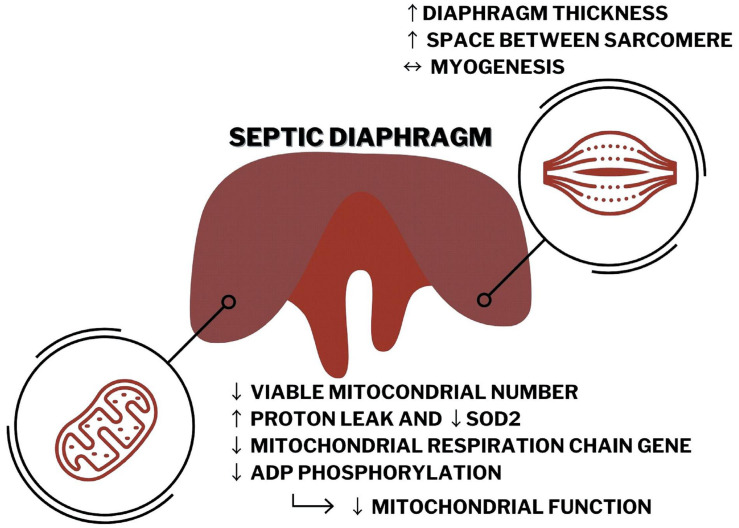

## Introduction

Sepsis is a life-threatening organ dysfunction associated with an imbalance host response to infection (Singer et al., 2016). Sepsis accounts for approximately half of hospital deaths in the United States, and patients usually need intensive care treatment (Thompson et al., 2017). The global sepsis incidence was estimated to be 48 million persons, and 11 million had septic-related deaths in 2017 (Rudd et al., 2020). These data suggest that 20% of worldwide death was related to sepsis (Rudd et al., 2020). Thus, sepsis is a significant public health concern in both high- and low-income countries.

Respiratory failure is a common cause for the admission of septic patients in intensive care units. Additionally, sepsis is a risk factor for developing acute respiratory distress syndrome (ARDS) (Thompson et al., 2017). ARDS accounts for approximately 10% of patients under intensive care treatment and more than 20% of patients under mechanical ventilation, and presents a high mortality rate (Bellani et al., 2016; Thompson et al., 2017). ARDS survivors have higher risks of developing skeletal-muscle weakness and exercise limitations up to 5 years after hospital discharge (Herridge et al., 2011; Thompson et al., 2017; Rudd et al., 2020).

Recent study suggests that respiratory muscles have an essential role in developing ventilatory failure, especially the diaphragm (Bloise et al., 2016; Penuelas et al., 2019). Mechanical ventilation itself can cause diaphragm weakness (Penuelas et al., 2019). However, recent findings demonstrated that diaphragm failure occurs before the mechanical ventilation intervention (Demoule et al., 2013; Supinski and Callahan, 2013; Supinski et al., 2018). Surprisingly, very few groups evaluated the diaphragm physiology during sepsis (Supinski and Callahan, 2013). Patients with the most severe diaphragm weakness presented the worst prognostic (Supinski and Callahan, 2013). Post-sepsis muscle weakness affects patients with no previous comorbidities and can impact life quality after hospital discharge for more than 6 months (Yende et al., 2016; Inayat et al., 2020).

Endotoxemia animal models demonstrated that bacterial lipopolysaccharide (LPS) signaling cascade could increase muscle proteolysis (Ono et al., 2020). Sepsis myopathy is also associated with increased proteasomal degradation, sarcomeric disorganization, and decreased insulin signaling in the peripheral muscle (Callahan and Supinski, 2009). Additionally, skeletal muscle response to sepsis differs according to the muscle fiber type (Owen et al., 2019). The slow-oxidative type I fibers are less affected by sepsis than the fast-glycolytic type II fibers (Owen et al., 2019). The murine septic model by cecal ligation and puncture (CLP) demonstrated decreased skeletal muscle cross-sectional area in the diaphragm and tibial anterior 24 h after surgery (Moarbes et al., 2019). However, the diaphragm returns to the control condition 96 h after CLP, while the tibial anterior cross-sectional area is still reduced compared with the control (Moarbes et al., 2019).

It is crucial to notice that the diaphragm is a continuous active skeletal muscle formed by almost equal amounts of type I and II fibers (Polla et al., 2004). Thus, the diaphragm physiology during sepsis can differ from peripheral muscle (Stana et al., 2017; Talarmin et al., 2017). Understanding these changes could help to treat the septic patients with respiratory failure better. Diaphragm weakness can be correlated with the mortality rate in intensive care units (Supinski et al., 2018). Our group recently demonstrated that severe untreated acute sepsis could reduce the mitochondrial number in the diaphragm and show unbalance in thyroid hormone signaling related to mitochondrial impairment in the diaphragm (Bloise et al., 2020). The mechanisms associated with diaphragm dysfunction during treated and more prolonged sepsis are still not well understood. In this study, we explored molecular pathways and morphological changes in the diaphragm and mitochondria during moderate sepsis to suggest the physiological routes that could disrupt diaphragm physiology.

## Materials and Methods

### Ethics Statement and Animal Experiment

All animals aged 4–5 weeks used were purchased from the Multidisciplinary Center for Biological Research Chronic – UNICAMP. Animals were acclimated and maintained at the Multiuser Biological Model Laboratory from the Carlos Chagas Filho Institute of Biophysics – UFRJ. The experiments were performed using 12–14 weeks old C57BL/6 male mice weighing 20–26 g. The conventional animals were housed in ventilated cages, 3–4 animals per cage in a 12-h light/dark cycle at a constant temperature (22°C). Water and chow (standard diet AIN93) were available *ad libitum*. CLP is the gold standard murine sepsis model (Laudanski, 2021). Sepsis was induced in the experimental group by CLP; control animals were sham-operated as described previously (Rittirsch et al., 2009; Bloise et al., 2020). In brief, after anesthetized with ketamine (100 mg/kg; Cristália, Itapira, Brazil) and xylazine (10 mg/kg; Syntec, Barueri, Brazil) intraperitoneally, the cecum was located, and below the ileocecal valve the cecal ligation was performed. Then, using an 18G needle, the cecum was perforated once between the ligation and its end. Sepsis severity was scored before any animal manipulation at 8 h after surgery and then every 24 h until 5 days after surgery (the score for measure sepsis severity description is given in Supplementary Material). Animals were treated with Meropenem (20 mg/kg, Aspen Pharmacare, Durban, South Africa) intraperitoneally 8, 24, 48, 72, and 120 h after surgery. Animals were euthanized under anesthesia with 1.5–2.0% of isoflurane (Isoforine, Cristália, SP, Brazil) 120 h after surgery. Diaphragms were collected, immediately frozen at liquid nitrogen, and stored at −70°C for the molecular biology analysis. The freshly dissected diaphragms were also collected for mitochondrial physiology measurement or immerse in 4% of paraformaldehyde for further microscopy analysis. The animal handling and euthanasia procedures were approved by the Federal University of Rio de Janeiro Animal Care Committee (CEUA-088/15).

### mRNA Isolation and qPCR

The total RNA from diaphragm samples (15 mg) was extracted using the TRIzol Reagent (Invitrogen, Carlsbad, CA, United States) and Macherey Nagel Kit (Macherey Nagel, Düren, DE) as described previously (Herridge et al., 2011). The cDNA synthesis was performed using the High Capacity cDNA Reverse Transcription Kit (Applied Biosystems, CA, United States) with 800 ng of total RNA according to the protocols of the manufacturer. After the cDNA synthesis, mRNA expressions were evaluated by qPCR using the HOT FIREPol^®^ Evagreen^®^ qPCR Supermix (Solis Biodyne, Denmark) and the Mastercycler Realplex system (Eppendorf, Germany). Table 1 contains the sequences of the primer pair. In summary, the following fiber profiles were evaluated: *Myhc7*, *Myhc4*, *Atp2a1*, and *Atp2a2*; the following myogenesis are investigated: *Myod1* and Myog; the following mitochondrial physiology are analyzed: *Ppargc1a*, *Opa1*, and *Dnm1l*; the following respiratory chains were investigated: *Ndufb8*, *Sdhb*, *Uqcrc2*, and *Cox4i1*; and the following reactive oxygen defenses are investigated: mitochondrial superoxide dismutase 2 (SOD 2; *Sod2*). Relative mRNA expression quantification was calculated using the standard curve method, and the expression level is related to the mean of the reference gene values (*Phctr* and *Pib* for diaphragm and *Vdac* for genes located in the mitochondrion). The best reference genes were chosen according to their *Cq* values and variances between the groups. PCR programs were as follows: denaturation 12 min 95°C, 40 cycles of 15 s 95°C, 30 s 60°C, and 30 s 72°C, following melting program. qPCR quality and genomic DNA contaminations were checked using Intron-spanning primers, reverse transcriptase-negative samples from cDNA synthesis, and melting curve analysis obtained from each reaction.

**TABLE 1:** Primer list.

**Gene**	**Primer sequences(forward top, reverse bottom)**	**GenBank accession no.**	**Amplicon size**	**Protein encoded (related function)**
*Phactr4*	ACTTTACACGCTACCATCGCCCAT AAGGGGAGCACAAGGACACG	NM_175306	190 bp	Phosphatase and Actin Regulator 4 (reference gene)
*Ppib*	GAGACTTCACCAGGGG CTGTCTGTCTTGGTGCTCTCC	NM_011149	253 bp	Peptidylprolyl Isomerase B (reference gene)
*Vdac*	GGGCTGACGTTTACAGAGAAG CTCATAGCCAAGCACCAGAGC	NM_001362693	240 bp	Voltage-Dependent Anion Channel 1 (mitochondrial reference gene)
*Ppargc1a*	CAATGAATGCAGCGGTCTA GTGTGAGGAGGGTCATCGTT	NM_008904.2	112 bp	Peroxisome Proliferator-Activated Receptor Gamma Coactivator 1-Alpha (PGC1α – a key regulatory factor of mitochondria biogenesis and function)
*Opa1*	TGGAAAATGGTTCGAGAGTCAG CATTCCGTCTCTAGGTTAAAGCG	NM_001199177.1 NM_133752.3	77 bp	OPA1 Mitochondrial Dynamin Like GTPase (promotes mitochondria fusion)
*Dnm1l*	TAAGCCCTGAGCCAATCCATC CATTCCCGGTAAATCCACAAGT	NM_001360010.1	77 bp	Dynamin 1 Like (promotes mitochondria fission)
*Ndufb8*	TGTTGCCGGGGTCATATCCTA AGCATCGGGTAGTCGCCATA	NM_026061	127 bp	NADH:Ubiquinone Oxidoreductase Subunit B8 (a subunit of the respiratory complex I)
*Sdhb*	AATTTGCCATTTACCGATGGGA AGCATCCAACACCATAGGTCC	NM_001355515	104 bp	Succinate Dehydrogenase Complex Iron-Sulfur Subunit B (a subunit of the respiratory complex II)
*Uqcrc2*	TCTCTGGAAAACTATGCTCCTCT AAATGTGAGGTTCCCAAGTTGT	NM_025899.2	95 bp	Ubiquinol-Cytochrome C Reductase Core Protein 2 (a subunit of the respiratory complex III - gene in the mitochondrion)
*Cox4i1*	TCCCCACTTACGCTGATCG GATGCGGTACAACTGAACTTTCT	NM_009941	149 bp	Cytochrome C Oxidase Subunit 4I1 (a subunit of the respiratory complex IV)
*Sod2*	CCAAGGGAGATGTTACAACTCAG GGGCTCAGGTTTGTCCAGAA	NM_013671	100 bp	Superoxide Dismutase 2 (mitochondrial defense against oxygen reactive species, converts superoxide into hydrogen peroxide and diatomic oxygen)

### Skeletal Muscle High-Resolution Respirometry

For the high-resolution respirometry (HRR), 3 mg of the diaphragm was dissected from the right area of the costal muscle domain. The tissue was washed in chilled BIOPS buffer (2.77 mM CaK_2_EGTA, 7.23 mM K_2_EGTA, 20 mM imidazole, 20 mM taurine, 6.56 mM MgCl_2_, 5.77 mM ATP, 15 mM phosphocreatine, 0.5 mM dithiothreitol, and 50 mM K-MES, pH 7.1). Then, it was permeabilized in cold BIOPS buffer supplemented with saponin (50 μg/ml) on ice for 40 min with gentle agitation. Permeabilized tissues were weighed, and samples were then transferred to chilled Mir05 buffer (110 mM sucrose, 60 mM K-MES, 0.5 mM EGTA, 3 mM MgCl_2_, 20 mM taurine, 10 mM KH_2_PO_4_, 20 mM K-HEPES, 1 g/L de bovine serum albumin (BSA) fatty acid free, pH 7.1). Tissues were maintained in Mir05 buffer for at least 10 min under mild agitation to remove saponin and metabolites and then transferred to measure oxygen consumption with an O2K (Oroboros Instruments GmbH, Innsbruck, Austria). Each O2K chamber was calibrated with Mir05 for at least 20 min before data acquisition.

Two sequential runs were performed for each tissue using one chamber for the sham tissue and the other for the CLP. In the next run, freshly permeabilized tissue was positioned in the opposite chambers to avoid errors associated with the equipment. The data of each run were treated as duplicate data for each animal. Thus, during further data analysis, the experimenter did not know which sample was from the diaphragms of sham and CLP. The background oxygen level from the tissue in Mir05 alone was recorded for at least 5 min, followed by substrate or drug administration. All substrate and drug administrations were titrated previously during test standardization. The oxygen consumption was recorded until stabilization after the addition of drugs or substrates as mentioned in the following sequence: 5 mM pyruvate and 5 mM malate (for recording basal respiration after substrate administration and stimulating complex I), 3 mM ADP (saturated ADP concentration to record coupled respiration), 10 μM cytochrome *c* (for assessing mitochondrial damage during permeabilization, O_2_ consumption increase above 5% was considered damage induced by the tissue process, and the data were excluded from the analysis), 10 mM succinate (coupled respiration associated with complex II), 2 μg/ml oligomycin (ATP synthase inhibitor – oxygen consumption related to proton leak or the uncoupled respiration, the dose was divided into two additions, but only the O_2_ consumption value after the second pulse was used for the analysis), 600 nM carbonyl cyanide-4-(trifluoromethoxy)phenylhydrazone (mitochondrial uncoupling agent, related to maximal respiration), and 10 mM potassium cyanide (KCN) (inhibitor of cytochrome *c* oxidase and non-mitochondrial oxygen consumptions). All O_2_ consumption raw data were subtracted from KCN values to discard non-mitochondrial oxygen consumption. After succinate, the subtraction of oxygen consumption values from the values after oligomycin gives us the oxygen consumption related to ADP phosphorylation (coupled respiration). The pmol O_2_ consumption was normalized by corresponding sample weight. The pyruvate, saponin, and KCN solutions were freshly made before each experiment.

### Transmission Electron Microscopy

Transmission electron microscopy (TEM) and mitochondrial quantification were performed as described previously (Bloise et al., 2020). In brief, diaphragms were immersed in 4% of paraformaldehyde for at least 48 h. The samples were then processed and analyzed (qualitatively and quantitatively) using a JEM1011 microscope (JEOL, Akishima, Tokyo, Japan). The fragments were washed in phosphate-buffered saline, fixed in 2.5% of glutaraldehyde sodium cacodylate (100 mM) buffer (pH 7.2) for 24 h, and washed three times in the same buffer. The tissue was post-fixed in 1% osmium tetroxide and sodium cacodylate buffer, dehydrated in acetone (30, 50, 70, 90, and 100%), and embedded in Poly/Bed812 resin (Ted Pella Inc., Redding, CA, United States). Ultrathin sections were contrasted with uranyl acetate-lead citrate and were collected in three grids with a distance of 7 μm between them. A total of 10 images were acquired from random fields in each grid (AMT XR80 CCD digital camera – Advanced Microscopy Techniques, Woburn, MA, United States; at 12,000× magnification, 70 nm). The total amount of mitochondria and the number of healthy and injured mitochondria were quantified in each electron micrograph. At least three images per tissue were analyzed. The qualitative evaluation consisted of analyzing the integrity of mitochondrial internal and external membranes, the mitochondrial morphology, and the matrix electron density. Healthy mitochondria have intact external and internal (mitochondrial cristae) membranes, with no signs of membrane disruption, continuous folds called crista, and a very electron-dense matrix (Supplementary Figure 1).

### *In vivo* Diaphragm Ultrasonography

Ultrasonography was performed and analyzed as described previously (Whitehead et al., 2016; Buras et al., 2019). The methodology was previously validated as a non-invasive methodology to access murine diaphragm function *in vivo* in a high-resolution ultrasonography system designed for mouse echocardiography, i.e., the Vevo 2100 (Fujifilm VisualSonics, Toronto, Canada) from the National Center for Structural Biology and Bioimaging (CENABIO-UFRJ, RJ, Brazil). The mice were anesthetized in an isoflurane chamber (1.5% isoflurane and 1.5% O_2_ L/min). Mice were fixated on the table by the four limbs taped onto the platform copper leads. Anesthesia was maintained using a nose-cone perfused with 1% isoflurane and 1.5% oxygen L/min. The chest and the abdomen of the mouse were shaved using depilatory cream to avoid any interference during the acquisition of the images. A small amount of ultrasound gel was applied between the animal skin and the probe (MS400) to guarantee proper image recording, as well as on the platform copper leads (Supplementary Figure 2). For diaphragm ultrasonography, each mouse was placed in a dorsal decubitus position, and the platform was tilted horizontally by 30° with the head of the mouse lower with respect to the feet. The probe was fixed transversally to the mid-sternal of the mouse at 180° with respect to the platform. Image acquisition was performed in mono-dimensional (M-Mode) and bi-dimensional (B-Mode). We obtained the following data: diaphragm thickness during inspiration and expiration, the thickness fraction, diaphragm excursion amplitude (excursion), inspiratory duration, peak-to-peak time, the excursion–time (E–T) index (product of diaphragm excursion and inspiratory period), and inspiratory duty cycle (quotient of inspiratory duration/peak-to-peak time). The thickness fraction is the measure of the thickness on inspiration (Ti) minus the thickness on expiration (Te) divided by the thickness on expiration; thus, the formula is (Ti - Te)/Te.

### Western Blot Analysis

The total protein was extracted from diaphragm fragments (±30 mg) in pH 7.4 RIPA lysis buffer (50 mM Tris–HCl, 150 mM NaCl, 1.0% Triton X-100, 0.1% SDS, 5 mM EDTA, 50 mM NaF, 30 mM sodium pyrophosphate tetrabasic, and 1 mM sodium orthovanadate) with complete protease inhibitor cocktail (Roche Diagnostics, Indianapolis, IN, United States) using the TissueLyser LT (QIAGEN). The total protein was quantified using a Pierce BCA Protein Assay Kit (Thermo Scientific) and was separated (10 μg) by SDS–PAGE in 8, 10, or 12% polyacrylamide gels and transferred onto a polyvinylidene difluoride membrane (Hybond-P 0.45 mm PVDF; Amersham Biosciences, United Kingdom) for 90 min at 0.25 A in a Bio-Rad wet system (Bio-Rad Laboratories, United States). The membranes were incubated with 5% of BSA (Sigma Life Science, United States) for 90 min at room temperature to block non-specific binding sites and then incubated overnight at 4°C with primary antibodies. The antibodies used were as follows: anti-Total OXPHOS Rodent WB Antibody Cocktail (Gel 12%; 1:1,000 dilution; cat. no. ab110413, Abcam, United Kingdom), OPA1 (Gel 10%; cat. no. 80471; 1:1,000 dilution, Cell Signaling Technology, Inc.), MyHC I (Gel 8%; 300 ng/ml; BA-F8; Developmental Studies Hybridoma Bank, United States), MyHC II A (Gel 8%; 300 ng/ml; SC-71; Developmental Studies Hybridoma Bank, United States), MyHC II B (Gel 10%; 300 ng/ml; BF-F3; Developmental Studies Hybridoma Bank, United States), and glyceraldehyde-3-phosphate dehydrogenase (GAPDH; 1:10,000 dilution; cat. no. 14C10, Cell Signaling Technology, United States) as the loading control. Alternatively, the membranes were then washed and incubated with peroxidase-labeled secondary anti-mouse antibody (1:10,000 dilution, cat. no. 62-6520, Invitrogen, United States) or anti-rabbit antibody (1:10,000 dilution, cat. no. 65-6120, Invitrogen, United States) for 150 min at room temperature. Immunoreacted proteins were detected by SuperSignal West Pico PLUS substrate or SuperSignal West Femto substrate (Thermo Fisher) in an ImageQuantLAS 4000 system, followed by densitometric analyses (GE Healthcare Life Sciences, United States). The Ponceau staining (Sigma Life Science, United States; cat. no. P7170) was used as the loading control in 8% gel. Data were corrected by the loading control and expressed relative to the Sham group.

### Statistical Analysis

The Kolmogorov–Smirnov test was used to test data normality and Grubbs’ test was used to remove outliers (alpha = 0.05). We used the Mann–Whitney *U* test (non-normally distributed data) or Student’s *t*-test (normally distributed data) to analyze the differences between groups. The Kaplan–Meier analysis was performed to investigate the survivor rate using Log-rank (Mantel-Cox) test to compare the mortality rates between CLP and sham groups. The results are shown as mean values ±SD of at least three animals per group. Statistical analysis was performed using the GraphPad Prism 5 software (GraphPad Software, Inc., San Diego, CA, United States). Differences were considered to be significant at *p* < 0.05. Symbols were used to describe *p* values < 0.05.

## Results

### Sepsis Clinical Score and Mortality

**FIGURE 1:**
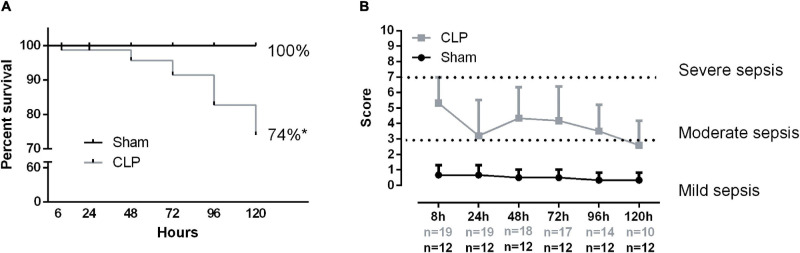
Sepsis clinical score and mortality. Animal survival rate **(A)**. The percentage compares the total live animal numbers at the beginning (0 h) and end of the experiment (120 h). Sepsis score time evolution **(B)**. The following variables evaluated the severity of sepsis: piloerection, alterations in gait, lethargy, alterations in respiratory rate, lacrimation, loss of grip strength, decreased body tone, respiratory difficulty after manipulation, lack of exploratory behavior, and body temperature alterations if present animal received 1 point. The sum of the total score reflected the severity of sepsis. Score 2–3 denotes mild sepsis, 4–7 moderated sepsis, 8–10 severe sepsis. Values under the *x*-axis time point represent the live animal number in each group, gray numbers represent CLP total animals, and black numbers represent the sham group. Black dots represent sham animals caused by surgical stress (*n* = 12), and gray squares represent CLP – cecal ligation puncture technique (*n* = 19 at 0 h and *n* = 10 at 120 h). Values expressed as mean ± SD, *p = 0.0007 chi-square, and **p = 0.0001 unpaired *t*-test compared with sham mice.

Sepsis was induced by CLP plus antibiotic treatment for 5 days. CLP mice presented a higher mortality rate compared with sham. Almost half of the septic mice did not survive (Figure 1A). Additionally, the survivor rate markedly decreases 72 h after the CLP. The majority of CLP animals developed moderate sepsis throughout the experiment (Figure 1B).

### Prolonged Sepsis Disturbs Diaphragm Ultrastructure but Does Not Function

**FIGURE 2:**
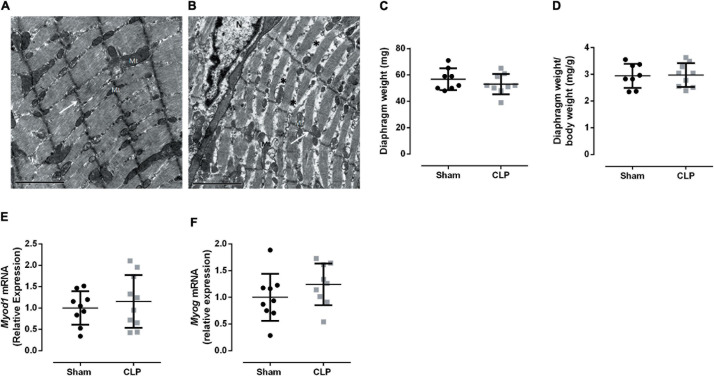
Prolonged sepsis disturbs diaphragm morphology. Transmission electron micrograph of Sham **(A)** and CLP **(B)** diaphragm fragments, white arrows indicate Z lines, * exemplify low electrodense areas between sarcomeres, *N* indicates the nucleus and Mt mitochondria, scales bars represent 2 μm. Diaphragm total weight **(C)** and related to animal body weight **(D)**. Expression of *Myod1* (expressed by activated satellite cells and myoblast; **E**), *Myog* (expressed by myocytes and early myotubes **F**). Black dots represent sham animals caused by surgical stress, and gray squares represent CLP – cecal ligation puncture technique. Values are expressed as mean ± SD, *p < 0.05, and **p < 0.005 compared with the sham group.

Cecal ligation and puncture-induced sepsis modified diaphragm ultrastructure presenting unallied and thinner sarcomeres, as can be observed by discontinuous Z lines and the low-electrodense spaces between sarcomeres (Figures 2A,B). These morphological changes could indicate a decrease in muscle mass. However, we did not observe a reduction in diaphragm weight (Figures 2C,D). Since the morphological change in the septic diaphragms could be related to an imbalance in tissue regeneration, the expressions of two myogenesis master regulators, namely, *Myod1* and *Myog*, were investigated, but no significant differences were observed between CLP and sham diaphragms (Figures 2E,F).

**FIGURE 3:**
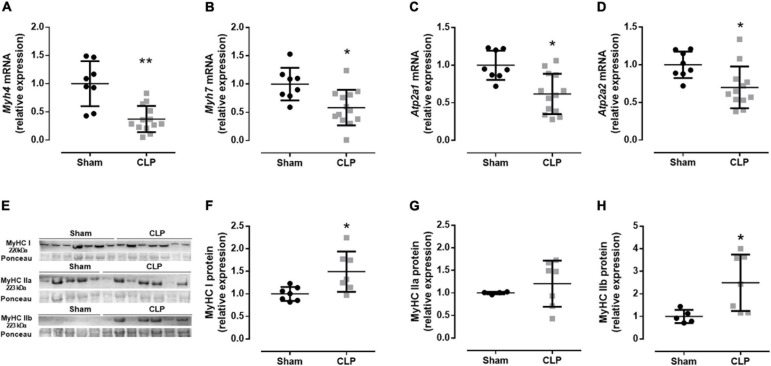
Moderate sepsis modulates fiber profile. mRNA expression of *Myh4* (encodes MHC IIb expressed in fast-glycolytic type II fiber; **A**), *Myh7* (encodes MHC I expressed in slow-oxidative type I fiber; **B**), *Atp2a1* (encodes SERCA1 expressed in fast-glycolytic type II fiber; **C**), and *Atp2a2* (encodes SERCA2 expressed in slow-oxidative type I fiber; **D**) in the diaphragm. Western blot representative figure for measuring MyHC I (fiber type I marker), MyHC IIa (fiber type IIa marker), and MyHC IIb (fiber type IIb marker; **E**). Relative protein expression of MyHC I **(F)**, MyHC IIa **(G)**, and MyHC IIb **(H)**. Black dots represent sham animals caused by surgical stress, and gray squares represent CLP – cecal ligation puncture technique. Values are expressed as mean ± SD, *p < 0.05, and **p < 0.005 compared with the sham group.

Then, we evaluated the expression fiber profile markers to better understand the physiological changes related to the diaphragm ultrastructural modification in CLP mice. The diaphragm is a mixed fiber and thus expresses both type I fibers, characterized by myosin I and SERCA 2a expression, and type II fibers, represented by myosin II (a, x, and b) and SERCA 1 expression (Greising et al., 2015). We investigated myosin and expression of SERCA isoforms from type I and type II fibers, *Myhc7* (myosin I, expressed in slow oxidative fibers), Atp2a2 (SERCA 2a), and *Myh4* (myosin IIb, the faster myosin expressed in the more glycolytic fiber), *Atp2a1* (SERCA 1), respectively. Related to the fiber I profile, we observed a decrease in myosin I and SERCA 2a mRNA expressions by 40 and 30% in septic animals, respectively, compared with sham. The myosin IIb expression and SERCA 1 decreased by 60 and 40% in the CLP diaphragm compared with the control expression, respectively (Figures 3A–D). The mRNA data suggested a reduction in type I and II fibers. However, the myosin protein expression demonstrated an increase in type I and IIb fibers and no modulation in type IIa fiber in the CLP diaphragm (Figures 3E–H). We observed an increase in MyHC I (49%) and IIb (140%) in the CLP diaphragm compared with sham (Figures 3E,F,H).

**FIGURE 4:**
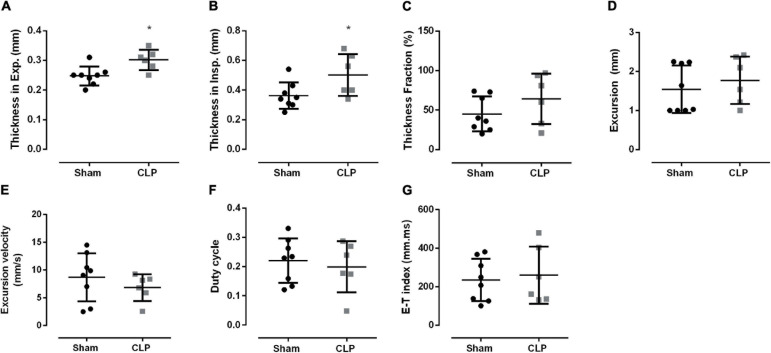
Diaphragm function is maintained during sepsis. Diaphragm thickness during expiration **(A)** and inspiration **(B)**; thickness fraction, an indication of contractability work, is the measure of the thickness on inspiration minus the thickness on expiration divided by the thickness on expiration **(C)**; excursion (movement amplitude, **D**); excursion velocity **(E)**, duty cycle (inspiratory duration/peak to peak, **F**), E–T index **(G)**. Black dots represent sham animals caused by surgical stress, and gray squares represent CLP – cecal ligation puncture technique. Values are expressed as mean ± SD and *p < 0.05 compared with the sham group.

Then, we investigated diaphragm function *in vivo* using ultrasonography (Figure 4). Although diaphragm mass did not change during sepsis (Figures 2C,D), we observed an increase in diaphragm thickness in inspiration (38%) and expiration (21%) in CLP compared with sham (Figures 4A,B). Thickness fraction, amplitude excursion, and excursion velocity did not change in CLP mice (Figures 4C–E). The other functional ultrasound data, i.e., duty cycle (the fraction of each breath spent during inspiration) and E–T index, did not change in CLP compared with control (Figures 4F,G).

### Sepsis Disrupted Diaphragm Mitochondrial Function

**FIGURE 5:**
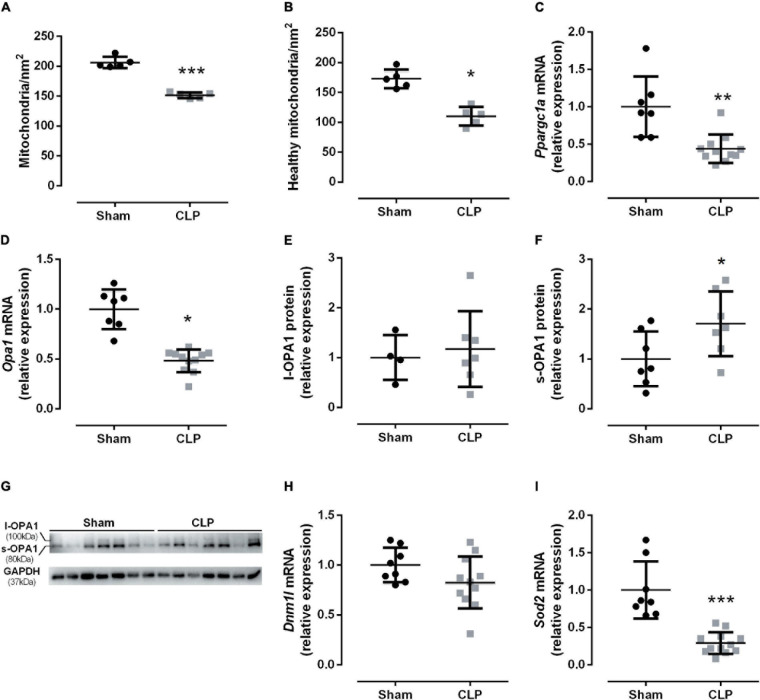
Sepsis disrupted diaphragm mitochondrial dynamics. Quantification of mitochondrial total **(A)** and healthy **(B)** quantity. The mitochondrial quality was assessed by the following factors: disrupting the mitochondrial membranes (injured mitochondria), the mitochondrial morphology: the preservation of the mitochondrial cristae, and the matrix electron density (healthy mitochondria), intact external membranes (healthy mitochondria), intact cristae and continuous folds (healthy mitochondria), and matrix electron density (injure mitochondria present low electron-dense matrix). mRNA expression of *Ppargc1a* (encodes PGC1α; **C**); *Opa1* (fusion marker; **D**); relative protein expression of the functional l-OPA1, 100 kDa isoform **(E)**, cleaved isoform s-OPA1, 80 kDa **(F)**, OPA1 and GAPDH (loading control) representative Western blot gels **(G)**. mRNA expression of *Dnm1l* (fission marker; **H**) and *Sod2* (encodes mitochondrial superoxide dismutase – SOD2; **I**). Black dots represent sham animals caused by surgical stress, and gray squares represent CLP – cecal ligation puncture technique. Values are expressed as mean ± SD, *p < 0.05, **p < 0.005, and ***p = 0.0002 compared with sham group.

The electron micrograph from Figure 2 suggested a decrease in the mitochondrial amount in the CLP diaphragm. The total and healthy mitochondria decreased by 35% in septic mice than sham-operated (Figures 5A,B). Then, we investigated the PGC1α gene expression (encodes by Ppargc1a), a key mitochondriogenesis stimulator. We observed that *Ppargc1a* expression decreased by half in the septic diaphragm (Figure 5C), suggesting that mitochondriogenesis was not triggered by the decrease in healthy mitochondria available in the septic diaphragm.

Subsequently, we investigated the fusion and fission dynamics by *Opa1* (a mitochondrial fusion marker) and *Dmn1l* (a mitochondrial fission marker) mRNA expression. In fact, we observed a 50% reduction in the fusion marker *Opa1* in septic diaphragms (Figure 5D). The functional long OPA1 isoform (l-OPA1) did not change between the groups (Figures 5E–G), but the non-functional small OPA1 (s-OPA1) protein level increased by 70% in the septic diaphragm (Figures 5E–G). The fission marker *Dmn1l* mRNA expression did not change between groups (Figure 5H). Additionally, we then investigated the antioxidant mitochondrial defense expression, namely, the superoxide dismutase 2 (Sod2). The diaphragm *Sod2* expression decreased by 70% in the CLP group compared with the control group (Figure 5I).

**FIGURE 6:**
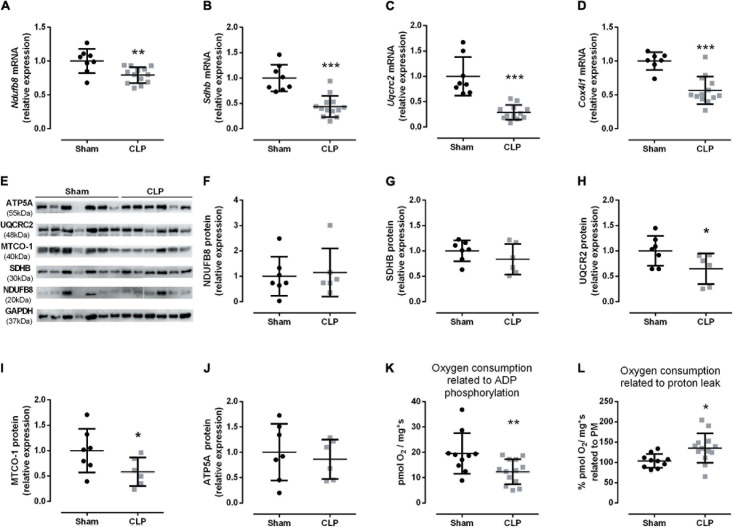
Sepsis impairs diaphragm mitochondrial function. Expression of complex I (*Ndufb8* – **A**); II (*Sdhb* – **B**); III (*Uqcrc2* – **C**), and IV (*Cox4i1* – **D**) in the diaphragm. Representative protein expression blot from complexes I–V **(E)**; protein quantification of complex I (NDUFB8 – **F**); complex II (SDHB – **G**); complex III (UQCR2 – **H**); complex IV (MTCO1 – **I)**; ATP synthase (complex V; ATP5A – **J**). Diaphragm HHR: oxygen consumption coupled to ADP phosphorylation (O_2_ consumption after succinate administration values minus O_2_ consumption after oligomycin administration values), the data values are an absolute measure of O_2_ consumption **(K)**; oxygen consumption related to proton leak **(L)**, values represent the percentage of O_2_ consumption after oligomycin administration related to pyruvate and malate O_2_ consumption values. Black dots represent sham animals caused by surgical stress, and gray squares represent CLP – cecal ligation puncture technique. Values are expressed as mean ± SD, *p < 0.05, **p < 0.005, and ***p < 0.001 compared with sham group.

The decrease in healthy mitochondrial amount (Figure 5) could suggest a reduction in oxidative phosphorylation function during sepsis. Therefore, we analyzed the respiratory chain complexes I–IV (*Ndufb8*, *Sdhb*, *Uqcrc2*, *Cox4i1*, respectively) mRNA expressions and protein levels. All gene expressions were reduced in the septic diaphragm group (Figures 6A–D). The protein levels of respiratory chain complexes III (UQCR2) and IV (MTCO-1) decreased by 36 and 42%, respectively, in the CLP group compared with the control (Figures 6E–J). These reductions could impair mitochondrial transmembrane electrochemical gradient and, thus, disrupt oxidative phosphorylation. Then, we investigated diaphragm oxygen consumption by HRR. After addition of pyruvate and malate, the basal respiration did not change between CLP and sham diaphragm (sham: 25.60 ± 2.34 pmol O_2_/mg^∗^s, *N* = 10; CLP: 21.35 ± 2.59 pmol O_2_/mg^∗^s, *N* = 13). Also, the maximal respiration measured after the addition of FCCP did not change (sham: 39.48 ± 3.85 pmol O_2_/mg^∗^s, *N* = 10; CLP: 34.38 ± 1.69 pmol O_2_/mg^∗^s, *N* = 13). However, we observed a 40% decrease in the oxygen consumption associated with ADP phosphorylation in septic mice (Figure 6K). Additionally, the oxygen consumption related to proton leak increased by 35% in septic diaphragms related to basal respiration (Figure 6L), but this absolute value did not change (sham: 26.40 ± 2.69 pmol O_2_/mg^∗^s, *N* = 10; CLP: 25.89 ± 2.22 pmol O_2_/mg^∗^s, *N* = 13).

## Discussion

In lung physiology, respiratory disruption caused by sepsis is well established (Bloise et al., 2016; Penuelas et al., 2019). However, recent findings suggest that sepsis also decreases peripheral skeletal muscle function (Owen et al., 2019). In fact, sarcomeric disorganization, increased protein degradation, and unbalancing energy production are present in peripheral muscle from critically ill patients (Callahan and Supinski, 2009). In contrast, the impact of sepsis on the diaphragm, the primary respiratory skeletal muscle, is not well understood. Herein, we demonstrated that antibiotic-treated moderate sepsis leads to decreased mitochondrial function and changes in diaphragm ultrastructure (Graphical Abstract).

Sepsis affects peripheral skeletal muscle mass differently depending on the fiber type (Bloise et al., 2016; Owen et al., 2019). The fast-glycolytic muscle mass decreases more than the slow-oxidative muscle mass during sepsis (Owen et al., 2019). Moreover, the gastrocnemius type II fiber showed sepsis-mediated atrophy, while the type I fiber demonstrated minor atrophy (Owen et al., 2019). Thus, the data from peripheral skeletal muscle may not be directly translated to the diaphragm, a mixed fiber muscle.

The Extensor digitorum longus (EDL), fast-twitch muscle, *ex vivo*-specific force decreases 1 week and 1 month in septic induced by cecal slurry compared with control mice (Owen et al., 2019). Moreover, the *ex vivo* diaphragm function was reduced 6 and 24 h after sepsis induction (Zolfaghari et al., 2015; Eyenga et al., 2021). This model resamples severe acute sepsis. Our sepsis animal model induces 5-day moderate sepsis (Figure 1). Additionally, our group demonstrated that acute and chronic systemic inflammation causes differential modulation in diaphragm physiology (Bloise et al., 2016). In this study, we showed that moderate sepsis reduces mitochondrial function and leads to diaphragm adaptation to maintain functional activity.

An increase in diaphragm thickness is associated with local inflammation or diaphragm work (Picard et al., 2012; Vivier et al., 2012). We did not observe inflammatory signs on the septic diaphragm on the electron micrographs. Thus, the augment in the diaphragm thickness in CLP mice might be related to an increase in diaphragmatic work. Therefore, our data suggest a compensatory adaptation during the septic progression.

Additionally, *ex vivo* data demonstrated that maximal isometric force generated was reduced in 24 h septic diaphragm strips compared with control fibers (Zolfaghari et al., 2015). The *ex vivo* force measure of the extensor digitorum longus (a muscle rich in type II fibers) demonstrated a decrease in specific force generation in 2 weeks and 1-month sepsis survivor mice (Owen et al., 2019). This group did not observe a change in soleus, gastrocnemius, tibial anterior, and extensor digitorum longus weight corrected to body weight (Owen et al., 2019). In our sepsis model, the diaphragm weight did not change as well. Although, the ultrasound data demonstrated an increase in the tissue thickness and no alteration in thickness fraction, indicating contractability work. Thus, we cannot discard that the augment in the diaphragm thickness is a physiological adaptation of the survivor septic mice that helped maintain the muscle work 120 h after CLP.

It is essential to notice that moderated sepsis manifestation represents most human septic cases (Paoli et al., 2018). Thus, understanding the diaphragm adaptations during prolongated sepsis models could help improve the knowledge associated with the impaired respiratory capacity observed in septic patients. Herein, we observed sarcomeric disorganization associated with modulating the fiber profile in the septic diaphragm (Figures 2, 3).

Endotoxemia induces muscle wasting *via* autophagy and proteolysis 48 h after LPS injection (Ono et al., 2020). Additionally, soleus (muscle rich in slow oxidative type I fibers) demonstrated a decrease in cross-sectional area of all type II fibers 4 days after sepsis (Owen et al., 2019). Autophagy and proteasome pathways were increased in the septic diaphragm (Stana et al., 2017). We observed an increase in the spaces between sarcomeres in the CLP diaphragm, suggesting tissue wasting or injury (Figures 2A,B). The expression of myogenic transcription regulators did not change in the septic diaphragm (Figures 2E,F). Suggesting there is not a regenerative process occurring at this time point in the septic diaphragm. However, the fiber profile was modulated in the CLP muscle. We observed a reduction in myosin I and IIb and SERCA1 and 2a mRNA expression (Figures 3A–D), while the myosin I and IIb protein expression increased (Figures 3E–H). During muscle plasticity, the mRNA and protein levels can be different (Andersen and Schiaffino, 1997). Septic diaphragm fiber size and cross-sectional area decrease, also protein degradation increase during the first 24 h after sepsis (Stana et al., 2017; Talarmin et al., 2017; Moarbes et al., 2019). However, these parameters are restored 96 h to 7 days after sepsis (Stana et al., 2017; Talarmin et al., 2017; Moarbes et al., 2019). Thus, our myosin mRNA and protein data suggest that the septic diaphragm is a transitional tissue. Probably, more prolonged sepsis could lead to a decrease in type I and IIb fibers.

During stress, the mitochondrial fusion process attenuates the damage caused by injured organelles (Gustafsson and Dorn, 2019). Thus, healthy and partially damaged mitochondria fuse to maintain overall function (Youle and van der Bliek, 2012; Gustafsson and Dorn, 2019). Herein, we demonstrated that septic diaphragms reduce the healthy mitochondrial amount. Additionally, the septic diaphragm decreased the *Opa1* mRNA and increased s-OPA1 protein isoform, which does not induce mitochondrial fusion (Wai et al., 2015; Figures 5D–G). Thus, our HRR data suggest that damaged mitochondria were not restored. Therefore, it can lead to a decrease in energy production. In fact, we observed a reduction in ADP phosphorylation in the diaphragm from the CLP group (Figure 6K). Moreover, the data from intercostal respiratory muscle from septic patients also decreased citrate synthase and complex I activity (Fredriksson and Rooyackers, 2007).

Pgc1α stimulates mitochondrial genesis and functions in skeletal muscle (Eisele and Handschin, 2014; Bloise et al., 2018). The Pgc1α expression upregulates during an increase in energy demand and intensification in ROS production (Eisele and Handschin, 2014). However, systemic inflammatory reactions can deregulate Pgc1α action in the peripheral skeletal muscle *via* NFκb signaling (Eisele and Handschin, 2014). Therefore, we investigated *Ppargc1a* expression (encodes Pgc1α) in the diaphragm during sepsis. We observed a decrease in *Ppargc1a* expression in the CLP diaphragm (Figure 5C). Pgc1α is a rapidly degraded coactivator associated with genomic and mitochondrial DNA, which is a crucial regulator of mitochondrial function, as oxidative phosphorylation, number, and mass (Villena, 2015). Skeletal muscle highly expresses Pgc1α, especially the slow type I fibers, associated with the adaptation to high energy demand (Villena, 2015). Thus, the reduction in *Ppargc1a* expression could be related to decreased mitochondrial energy production observed in the septic diaphragm (Figure 6K). Additionally, Pgc1α induces the expression of detoxifying ROS enzymes such as Sod2 (Eisele and Handschin, 2014). Septic animals also decreased the expression of *Sod2* (Figure 5I).

Additionally, the diaphragm seems more resistant to oxidative damage than limb muscle with no increase in lipid peroxidation (Talarmin et al., 2017). However, it did not present a significant change in the SOD protein level (Talarmin et al., 2017). Another group demonstrated that acute sepsis increased H_2_O_2_ production in the diaphragm (Eyenga et al., 2021). Reduction in *Sod* expression observed herein can be a consequence of the decrease in *Pgc1*α observed (Figures 5, 6). However, we cannot discard that other factors might be involved in the antioxidative enzyme control, since we did not evaluate the antioxidative defense enzymatic activity and oxidative damage (Shan et al., 2015; Ahmed et al., 2017).

The increased unhealthy mitochondrial amount (Figure 5B) could be one of the causes of tissue damage. CLP diaphragm presented an increase in proton leak (Figure 6L). The proton leak is a cellular defense from the increased ROS production (Zhao et al., 2019). Thus, it could be an adaptation process to handle septic mitochondrial damage. We previously demonstrated that acute sepsis (24 h CLP) decreases the critical cellular antioxidative protein expression in septic diaphragms (Bloise et al., 2020). Therefore, the disruption in mitochondrial physiology induced by CLP demonstrated here could be one factor that leads to diaphragm dysfunction during sepsis.

Whitehead et al. (2016) demonstrated a positive correlation between diaphragm excursion amplitude measurement by ultrasound and diaphragm-specific force measure by traditional *ex vivo* force measurements (Whitehead et al., 2016). The E–T index is associated with forcing generation (Palkar et al., 2018). In the sepsis diaphragm, the E–T index did not change (Figure 4G). Also, in the duty cycle, another functional data (Buras et al., 2019), the diaphragm contraction parameters seem not to be affected by sepsis (Figure 4E). Thus, besides the decrease in the mitochondrial function, the diaphragm appears to be adapted to maintain the contractile work in the prolonged septic group. It is crucial to notice that our data are related to the survivor group. Therefore, we cannot assume that the same modulation occurred in the non-survivor septic animals. We have demonstrated previously that 24 h after CLP, the mitochondrial function is already decreased. However, the diaphragm sarcomeric organization is preserved (Bloise et al., 2020). Herein, the diaphragm function did not change; however, the sarcomeric organization seems impaired (Figure 2B). Thus, in the survivor septic group, diaphragm ultrastructural disorganization and decreased mitochondrial function were not enough to impair the primary respiratory muscle contraction. Furthermore, the diaphragm E–T index is used in clinical particles to guide the extubation time of patients. Hence, it is important to notice that a normal diaphragm contraction function may not reflect restored diaphragm morphology.

Herein, we demonstrated that 5 days after moderate sepsis induction led to mitochondrial damage and diaphragm morphological changes. Additionally, we observed disruption in diaphragm fiber ultrastructure. These changes might be associated with diaphragm failure during sepsis conditions.

## Data Availability Statement

The raw data supporting the conclusions of this article will be made available by the authors, without undue reservation.

## Ethics Statement

The animal study was reviewed and approved by Federal University of Rio de Janeiro Animal Care Committee.

## Author Contributions

TO, AS, NR, and FB performed and designed the experiments and analyzed and interpreted the data. CA, JS, NB, and JW performed the experiments. PS and PR interpreted the data and wrote the manuscript. Wd-S designed the experiments, supervised the data acquisition, wrote the manuscript, and interpreted the data. TO-C and FB conceived and designed the experiments, supervised the data acquisition, interpreted the data, and wrote the manuscript. All authors contributed to the article and approved the submitted version.

## Conflict of Interest

The authors declare that the research was conducted in the absence of any commercial or financial relationships that could be construed as a potential conflict of interest.

## Publisher’s Note

All claims expressed in this article are solely those of the authors and do not necessarily represent those of their affiliated organizations, or those of the publisher, the editors and the reviewers. Any product that may be evaluated in this article, or claim that may be made by its manufacturer, is not guaranteed or endorsed by the publisher.
