# Supplementary material for: Sepsis Disrupts Mitochondrial Function and Diaphragm Morphology
Source: Front Physiol. 2021 Sep 7;12:704044. doi: 10.3389/fphys.2021.704044 (PMC8452856; doi:10.3389/fphys.2021.704044)
Supplement: Supplementary file 1 [file Data_Sheet_1.docx]

**Supplementary information**

**The score for measure sepsis severity**

Sepsis severity assessment includes the analysis of the following variable: piloerection, alterations in gait, lethargy, alterations in respiratory rate, lacrimation, loss of grip strength, decreased body tone, respiratory difficulty after manipulation, lack of exploratory behavior, and body temperature alterations. The animal received 1 point per variable present. The sum of the total score reflected the severity of sepsis. Score 2-3 points mild sepsis, 4-7 moderated sepsis, 8-10 severe sepsis. Sepsis severity was scored at eighth hours, 24h, 48h, 72h, 96h, and 120h after surgery. Although the high mortality rate (53%), most survivor animals scored as moderate sepsis during the all-time analyzed.


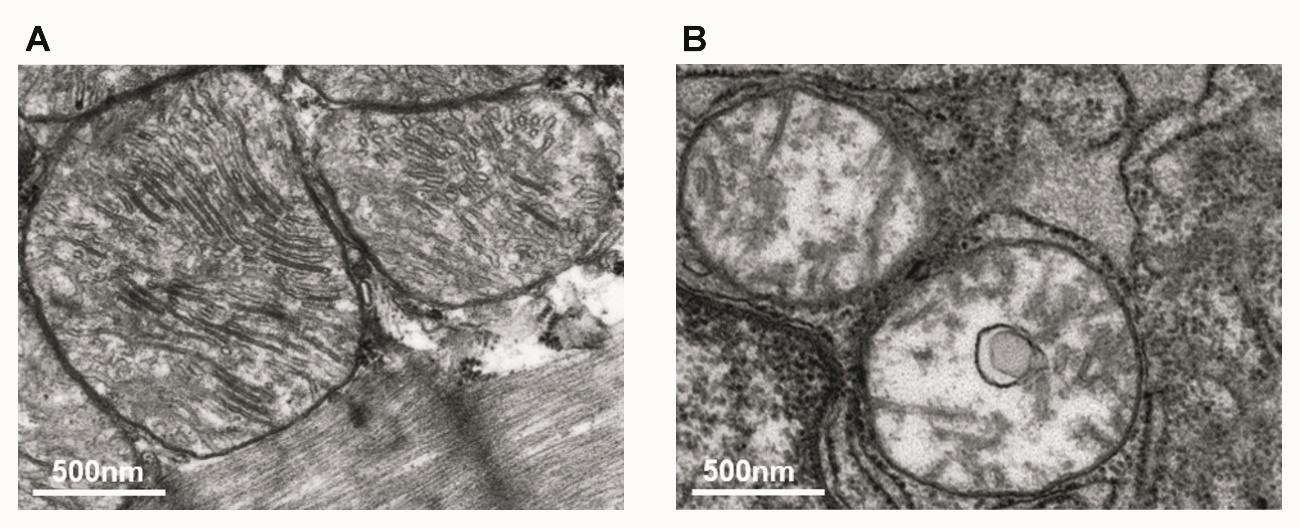


**Supplementary figure 1. Characterization of health and injured mitochondria.** TEM electromicrographs of two health mitochondria presenting external membrane integrity and internal membrane (cristae) integrity demonstrated by high electrodense and continuous membranes, no signs of membrane disruption, continuous folds crista, and a very electron-dense matrix (A). TEM electromicrographs of two injured mitochondria, presenting low electrodense matrix, low electrodense internal membrane (cristae), and disorganized cristae (B).


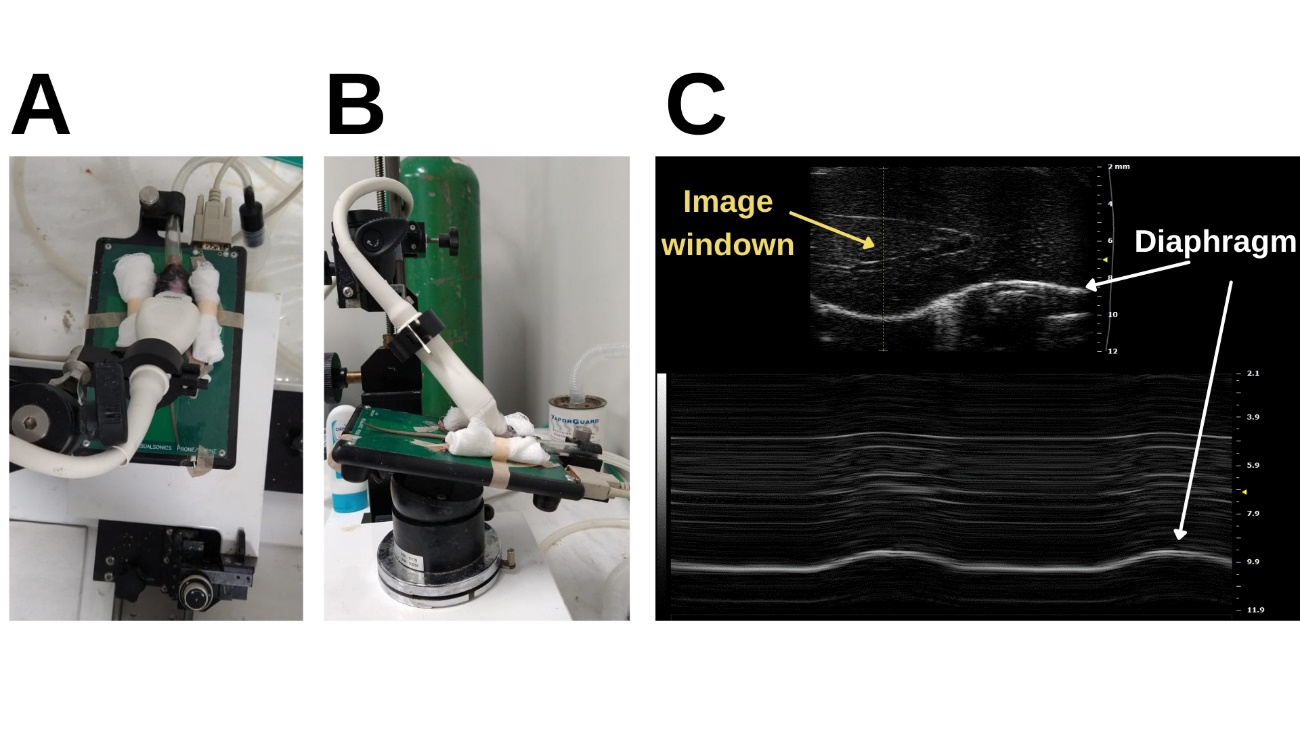


**Supplementary figure 2. Animal setup at the high-resolution ultrasonography system Vevo 2100.** Stage step and animal position (A, B), representative image window from ultrasonography, on the top location diaphragm position and M-mode to acquire diaphragm data (yellow dotted line, and yellow arrow), on the bottom representative trace of diaphragm movement, white arrows identified the diaphragm (C).
